# Successful Treatment of Mesotherapy Induced Granuloma Annulare With Baricitinib

**DOI:** 10.1111/jocd.70162

**Published:** 2025-04-01

**Authors:** Li Wang, Shang Shen Chen, Ming Yuan Xu, Ye Qiang Liu, Jun Ting Tang

**Affiliations:** ^1^ Department of Dermatology Shidong Hospital Affiliated to University of Shanghai for Science and Technology Shanghai China; ^2^ Huan Kui College of Nanchang University Nanchang China; ^3^ Department of Dermatopathology Shanghai Skin Disease Hospital, Tongji University School of Medicine Shanghai China; ^4^ Department of Dermatology and Venerology First Affiliated Hospital of Kunming Medical University Kunming China

**Keywords:** Baricitinib treatment, granuloma annulare, mesotherapy

Dear editor,

Mesotherapy is a dermatological treatment that involves injections into the dermal layer to achieve effects such as pore reduction, enhanced hydration, improved skin tone, and wrinkle removal [1]. Although generally regarded as safe, its widespread use and the absence of standardized procedures have led to various adverse reactions, including granuloma formation, infections, fat necrosis, and abscesses. Systemic corticosteroids are considered the primary treatment; however, consensus is lacking regarding alternative therapeutic options [2]. Granuloma annulare is a benign inflammatory condition associated with various comorbidities, including thyroid disease, malignancy, trauma, diabetes mellitus, and HIV infection [2]. While systemic corticosteroids are effective in managing granuloma annulare, the condition frequently recurs following the cessation of treatment. Baricitinib, an inhibitor of the Janus kinase/signal transducers and activators of transcription (JAK/STAT) pathway, exhibits promising therapeutic effects on granuloma annulare (GA). In this context, we report on a patient with Mesotherapy‐induced Granuloma Annulare whose lesions significantly improved with the administration of the JAK1/2 inhibitor, Baricitinib.

A 32‐year‐old female exhibited diffuse red papules and nodules on her face following mesotherapy injections intended for facial aesthetic enhancement with one‐week history. The injectant, hyaluronic acid, was procured by the patient and administered using a handheld needle. Verification of the substance's nature and chemical composition was not feasible. A skin biopsy was obtained from a singular lesion on the forehead.

Upon physical examination, diffuse papules and subcutaneous nodules were observed bilaterally on the cheeks, forehead, and lower jaw, aligning with the mesotherapy injection sites (Figure 1a,b). Dermoscopic examination revealed multifocal yellow‐orange areas along with several globular white regions, indicative of the histologic subtype of granuloma (Figure 1d). Histopathological examination showed accumulation of palisading granuloma, which was characterized by histiocytes and lymphocytes surrounding irregular zones of altered collagen and deposited mucus in the dermis; no obvious foreign body deposition was found (Figure 1e,f). Positive for Alcian blue staining (Figure 1g). These findings supported a diagnosis of granuloma annulare, grounded on characteristic clinical and pathological features.

**FIGURE 1:**
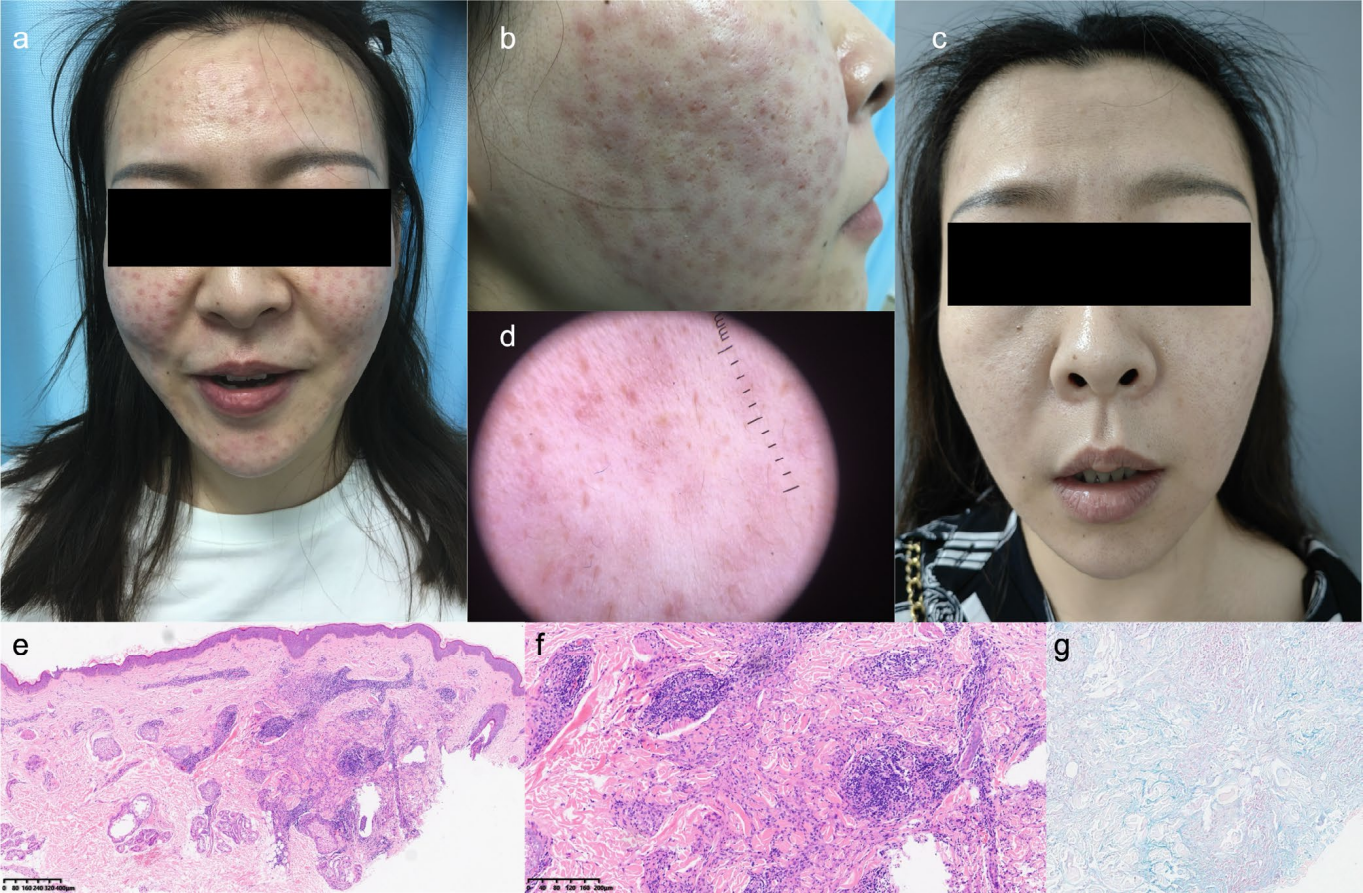
(a, b) Diffuse papules and subcutaneous nodules were observed bilaterally on the face, aligning with the mesotherapy injection sites. (c) One month after treatment with Baricitinib, the rash subsided. (d) Dermoscopic examination revealed multifocal yellow‐orange areas along with several globular white regions, indicative of the histologic subtype of granuloma. (e, f) Histopathological showed presence of lymphocytes, multinucleated giant cells, and histiocytes infiltrating and clustering around denatured collagen bundles and deposited mucus in the dermis. (g) Positive for Alcian blue staining.

The initial treatment regimen included doxycycline, administered orally at 100 mg twice daily for 1 month, and intramuscular administration of 7 mg betamethasone was performed once monthly, totaling two injections. However, these measures resulted in minimal improvement. Subsequently, the patient commenced treatment with oral baricitinib at a dose of 2 mg/day. A marked reduction in lesions was noted 1 month post‐initiation, with near‐complete resolution observed after 3 months (Figure 1c). No relapse was detected during a 4‐month follow‐up period following discontinuation of baricitinib.

## 1 Discussion

At present, there are more reports about foreign body granuloma, advanced granuloma, and infectious granuloma caused by cosmetic injection. The pathological examination of our case revealed a granulomatous reaction, with alcian blue staining positivity indicative of substantial mucin deposition. Consequently, the histopathological features were more aligned with a granuloma annulare pattern. However, the pathogenesis of mesotherapy‐induced granuloma annulare (GA) remains poorly understood. The patient provided a history of injected substance mainly as hyaluronic acid. Hyaluronic acid is widely recognized as a safe dermal filler; however, it can occasionally lead to the formation of late granulomas. Mr. Moran proposes that the generation of late granulomas may be linked to immune responses to residual bacterial cell wall components, such as lipoteichoic acid (LTA), present in the hyaluronic acid. Another contributing factor could be the degradation products of hyaluronic acid itself, which might initiate this inflammatory response. Additionally, in certain instances, the development of chronic nodules and granulomatous inflammation following filler injections can be traced back to bacterial, fungal, polymicrobial, or viral infections. This highlights the complex etiology behind inflammatory reactions to dermal fillers and underscores the need for thorough sterility and monitoring post‐injection to mitigate such risks [3, 4]. This generally takes place at the time of initial filler injection. However, there was no report about the mechanism research of mesotherapy‐induced granuloma annulare (GA). We hypothesize that the formation of granulomatous inflammation following hyaluronic acid injections in mesotherapy is influenced not only by the body's inability to metabolize hyaluronic acid but also by a variety of other factors. These include the molecular weight and osmotic pressure of hyaluronic acid, the physicochemical properties of its degradation products, the patient's immune status, and the level of bacterial contamination at the time of injection, or deep injection leads to malabsorption. Each of these factors potentially contributes to the complex pathophysiology of granulomatous inflammation in response to dermal fillers, underscoring the need for comprehensive pre‐injection assessments and stringent.

A pivotal study by Min et al. demonstrated notable elevations in interleukin‐4 (IL‐4) levels, T‐helper 2 (Th2) markers, and Janus kinase 2/3 (JAK2/3) in the lesion skin of GA patients compared to controls [5]. These findings suggest a notable immune dysregulation in GA pathophysiology. Additionally, the efficacy of JAK inhibitors, such as tofacitinib and upadacitinib, in treating GA lesions has been well documented [6]. Notably, Kasperkiewicz et al. reported that Baricitinib, a JAK1/2 inhibitor, targets the downstream signaling of IFN‐γ by inhibiting the JAK1/2‐STAT1 pathway. IFN‐γ, which is primarily secreted by Th1 cells, plays a pivotal role in macrophage activation and granuloma formation [7]. Given the pathological characteristics of the case and the suboptimal response to glucocorticoid therapy, we opted for a broader‐spectrum JAK1/2 inhibitor as part of the treatment strategy. To our knowledge, the case presented herein is the first instance of GA caused by mesotherapy successfully treated with Baricitinib, indicating a promising direction for future therapeutic strategies.

## Consent

The authors attest to obtaining written patient consent for the publication of recognizable patient photographs or other identifiable material, with the understanding that this information may be publicly available.

## Conflicts of Interest

The authors declare no conflicts of interest.

## Data Availability

The data that support the findings of this study are available from the corresponding author upon reasonable request. IRB has approved the study or exempted it from review.
